# Metabolic Syndrome and cognitive decline in the elderly: A systematic review

**DOI:** 10.1371/journal.pone.0194990

**Published:** 2018-03-26

**Authors:** Naima Assuncao, Felipe Kenji Sudo, Claudia Drummond, Fernanda Guarino de Felice, Paulo Mattos

**Affiliations:** 1 Memory Clinic, D’Or Institute for Research and Education, Rio de Janeiro, Brazil; 2 Institute of Biomedical Sciences–Morphological Sciences Program, Federal University of Rio de Janeiro, Rio de Janeiro, Brazil; 3 Department of Psychology, Pontifical Catholic University of Rio de Janeiro, Rio de Janeiro, Brazil; 4 Department of Speech and Hearing Pathology, Federal University of Rio de Janeiro, Rio de Janeiro, Brazil; 5 Institute of Medical Biochemistry Leopoldo de Meis, Federal University of Rio de Janeiro, Rio de Janeiro, Brazil; 6 Department of Psychiatry and Forensic Medicine, Institute of Psychiatry, Federal University of Rio de Janeiro, Rio de Janeiro, Brazil; Nathan S Kline Institute, UNITED STATES

## Abstract

**Background:**

Metabolic Syndrome (MetS) refers to a cluster of metabolic disturbances which is associated with increased risk for vascular and degenerative conditions in general population. Although the relationship between vascular risk factors and dementia is undisputable, additional hazard for cognitive decline in older population with concurrent metabolic disorders still waits to be demonstrated. The present review aims to analyze data on MetS and risk for cognitive decline in elderly persons.

**Methods:**

Database searches were performed in Medline, ISI and PsycINFO for articles assessing cognitive performances of older subjects with MetS.

**Results:**

Of a total of 505 studies, 25 were selected for the review. Risk of selection biases was identified in all the studies. Although all articles followed recognized diagnostic recommendations for MetS, minor criteria modifications were detected in most of them. Hyperglycemia was consistently associated with impaired cognitive performances in older individuals, but the role of MetS for cognitive decline and for the onset of dementia showed heterogeneous results.

**Discussion:**

Current available data in the literature concerning the impact of MetS on the cognition of older population is inconclusive and based on inconsistent evidence. Differential effects of individual MetS components and factors associated with the age of the sample may have accounted for divergent findings among articles, but larger and higher quality studies in this field are still needed.

## Introduction

Metabolic Syndrome (MetS) refers to a cluster of metabolic disorders that together are associated with a higher risk of cardiovascular disease and mortality [1]. It was first described in 1988 by G. M. Reaven, who reported that concurrent metabolic conditions, such as systemic arterial hypertension, impaired glucose tolerance (IGT), increased plasma triglyceride and decreased high-density lipoprotein (HDL) cholesterol concentration, might play a role in the onset of coronary artery disease [2]. Many studies have demonstrated that MetS is highly prevalent among adults, with rates in the male population ranging from 7% to 34%, and from 5% to 22% in adult women [3]. Moreover, evidence suggested that prevalence of MetS may increase with age, but epidemiology of this condition among elderly populations remains understudied [4].

Although the association between MetS and cardiovascular risk has long been established, there are several shortcomings in the available literature when it comes to the elderly population. Challenges to assess MetS in older subjects include the lack of universally applied diagnostic criteria and divergences among clinical guidelines for the recommended cardiovascular targets for this age group [5,6]. As indicated in previous studies, those issues account for a great variability of findings among studies [6]. Furthermore, questions linger as to whether MetS is a valid construct for older subjects, since some authors have suggested that the overall prognostic value of MetS in this group may not be greater than that of the single risk factors taken separately [1,6].

Moreover, a large amount of evidence indicated that cardiovascular and cerebrovascular disease may share the same risk factors [7]. Chronic metabolic insult may lead to arteriolosclerosis and hyalinosis on the cerebral small vessels, which can result in white matter damage and cognitive dysfunction [8]. The concept of “vascular cognitive impairment”—the continuum of cognitive deficits and dementia due to cerebrovascular disease–has been widely accepted as an important cause of cognitive impairment in the older population [9]. Most recently, investigations suggested that vascular risk factors may also contribute to the onset of sporadic Alzheimer’s Disease (AD) [10].

Although the presence of metabolic disturbances may increase the risk for cognitive decline and dementia, the association of MetS, as defined by current consensus guidelines, and cognitive impairment still waits to be established. Thus, the present review aimed to investigate a possible association between MetS and the risk of cognitive impairment.

## Methods

### Search strategy and selection criteria

On July 7^th^ 2017, database searches were performed in Medline, ISI Web of Knowledge and PsycINFO, using the following strategy: "metabolic syndrome" OR "plurimetabolic syndrome" OR "syndrome x" AND "cognition" OR "cognitive decline" OR "cognitive deficit" OR "dementia" OR "alzheimers disease" OR "mild cognitive impairment" AND "elderly" OR "older" OR "aged" OR "senior". No limits were placed on publication dates or fields.

Inclusion criteria were: (1) original articles which dealt with MetS in subjects aged ≥ 60 years old and (2) studies in which cognitive performance, assessed through neuropsychological tests was an outcome. Articles were excluded if one or more of the following features was identified: (1) middle-aged adults (<60 years old) were included in the same group as elderly individuals, so that conclusions regarding cognitive performances of older subjects could not be drawn; (2) studies did not apply the MetS construct, defined as a collection of clinical conditions and habits which may confer increased risk for vascular disease; (3) studies were written in languages other than English, Spanish, French or Portuguese; and (4) studies consisted of posters, conference papers, reviews, case reports or essays. Review articles and references from selected articles were searched for additional papers.

### Study selection, data collection and synthesis of the results

Screening of the retrieved articles was assessed by two independent authors (N.A. and F.K.S.). Data extraction was performed independently and divergences on the eligibility of papers were discussed with the whole team.

Information was extracted from each included paper on: (1) characteristics of the sample (including number of participants, age, schooling, gender and ethnicity); (2) design of the study (longitudinal or cross-sectional); (3) inclusion and exclusion criteria; (3) criteria applied for the diagnosis of MetS; (4) cognitive tests; (5) summary measures (e.g., relative risk, odds-ratio, hazard ratio, difference in means etc.) and (6) other relevant variables used in the studies (e.g., clinical and laboratory variables). Those variables were employed to compare results from the selected articles.

### Assessment of risk of bias

The quality of the selected studies was appraised through the Newcastle-Ottawa Quality Assessment Scale for Cohort Studies [11] and the STROBE Statement Checklist for cross-sectional studies [12].

The Newcastle-Ottawa Quality Assessment Scale for Cohort Studies includes sections addressing risk of selection, comparability of the groups and outcome biases. The STROBE statement comprises a checklist of items that should be included in cross-sectional studies—as for instance, the adequacy of the title and abstract, the rationale for the investigation being reported, the methods for the inclusion of participants, the selection of variables, the appropriateness of the statistical analyses and the description of the outcomes.

## Results

**Fig 1:**
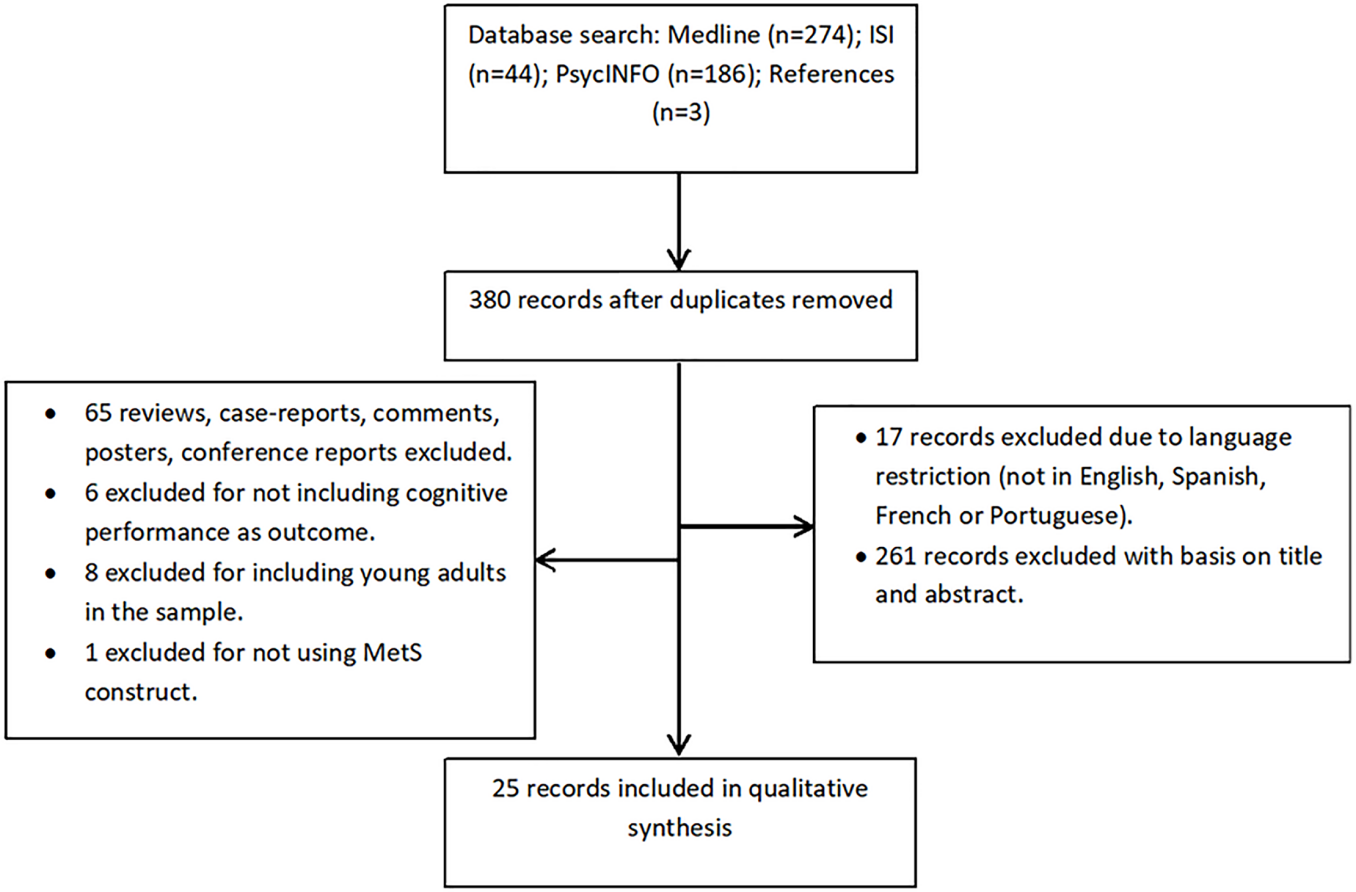
Stages of data search and selection.

A total of 507 articles were retrieved from the database and the reference searches. Of those, 25 papers fulfilled the inclusion criteria for qualitative analysis (Fig 1). The PRISMA Statement was applied for the preparation of this review (S1 Table).

### Sample

Overall, 28,646 elderly participants were included in the selected samples. Most of the studies were conducted in community settings, whereas 8 comprised convenience samples drawn from medical or nursing care facilities [13–20]. Educational level was generally low among studies; 7 of which included a significant number of subjects with formal education between 0 to 3 years [17,21–27], whereas 2 studies did not acknowledge the schooling of the sample [18,28].

Most of the studies assessed non-demented elderly subjects. One article, however, compared AD individuals with and without MetS [20], while other examined effects of MetS on healthy elderly controls and mild AD subjects [18]. Limits for age within the older range were predetermined for the recruitment of participants in some of the papers: Katsumata et al. (2012) chose to assess only residents above 80 years old from a town in Okinawa, Japan [29]; Liu et al. (2013) longitudinally evaluated males above 75 years old [28]; Van den Berg et al. (2007) followed a sample annually, starting at 85 until 90 years old [21]; the oldest-old subjects (above 85 years old) were also consecutively evaluated by Harrison et al. (2015) [26] and Viscogliosi et al. (2017) [27]. Moreover, some studies have restricted the selection of participants by gender: Liu et al. (2013) [28] and Viscogliosi et al. (2017) [27] assessed only older men, while elderly women were evaluated in the article authored by Komulainen et al. (2007) [30].

### Quality of studies

Potential selection bias was identified in all the elected studies. Restricting the sample for specific age clusters [21,23, 26–29], ethnic groups [13, 31], gender [27, 28, 30] or clinical and functional status [14, 15, 17–20, 24, 32–35] may have compromised the generalization of the results. Response rate at baseline was lower than 70% in 3 of the studies [25, 27, 30], whereas other did not acknowledge the baseline participation rate [36]. Issues related to the comparability among groups were detected in some articles due to significant differences in age [25, 37], gender [13, 20, 23, 24, 25, 27, 32, 33, 36], schooling [13, 21–25, 25, 33, 36], rates of institutionalized participants [21], presence of depressive symptoms [32] and ethnic composition [33, 36]. Risk of outcome bias was found in one of the studies, in which the follow-up lasted less than one year [16].

### Diagnosis

The diagnosis of MetS was based on the National Cholesterol Education Program’s Third Report of the Expert Panel on Detection, Evaluation, and Treatment of High Blood Cholesterol in Adults (NCEP-ATP III) criteria in 88% of the studies [38]. Recommendations for the diagnosis of MetS according to this group comprised the detection of 3 or more of the following: (1) abdominal obesity (waist circumference > 102 cm in males or > 88 cm in females); (2) increased plasma triglyceride level (≥150mg/dL); (3) low high-density lipoprotein cholesterol (HDL) level (<40mg/dL in males or <50mg/dL in females); (4) high blood pressure (systolic blood pressure ≥130mmHg or diastolic blood pressure ≥85mmHg); and (5) high fasting plasma glucose (≥100mg/dL) [38]. Some modifications in the original criteria were observed in most of the studies. Inclusion of subjects under pharmacological treatment for systemic arterial hypertension, dyslipidemia and diabetes mellitus was generally accepted as alternatives for the need of identifying high blood pressure, high triglyceride, low HDL levels or high fasting glucose during clinical and laboratory assessment [13,14,17, 20–24, 26, 28, 29, 32, 33, 35, 37]. Specifically, a few studies included the use of antihypertensive and antidiabetic medications, but not of lipid-lowering drugs, as indicators of metabolic risk [15, 16, 18, 27, 34, 36]. Other adaptations were the use of body mass index instead of the waist circumference [31, 32, 29, 35] and the replacement of fasting blood glucose for non-fasting glucose levels (impairment defined as glucose levels above 7.8 mmol/L) [21], glycated hemoglobin (HbA1c) level (≥ 5.4%) [29] or fructosamine levels (≥ 0.247 mmol/L) [13]. Modification on the desired target for high blood pressure among older persons (≥ 160x90 mmHg) was proposed by Dik et al. (2007) [13]. Different cutoffs for the diagnosis of central obesity were used in studies with specific ethnic populations; for instance: waist circumference ≥ 90 cm for East Asian males and ≥ 80 cm for East Asian females [24, 28, 37] or body-mass index ≥ 25 kg/m^2^ for Japanese population of both genders [29]. One of the studies distinguished subjects with diabetes (fasting glycemia ≥126 mg/dL or nonfasting glycemia ≥200 mg/dL or antidiabetic medication) from those with elevated fasting glycemia without diabetes (fasting glycemia ≥110 mg/dL) [25].

The International Diabetes Federation (IDF) criteria were used in two of the studies [19, 31]. Detection of MetS was based on the presence of obesity (body mass index > 30 kg/m^2^ or waist circumference ≥94 cm for Europeans, Sub-Saharan African, Eastern Mediterranean or Middle East males and ≥ 80 cm for European, Sub-Saharan African, Eastern Mediterranean or Middle East females; ≥90 cm for South Asian, Central or South American or Chinese males and ≥80 cm for South Asian, Central or South American or Chinese females; ≥85 cm for Japanese males and ≥90 cm for Japanese females) and 2 or more of the following: (1) increased plasma triglyceride level (≥150mg/dL) or medication therapy for this condition; (2) reduced HDL level (<40 mg/dl in males and <50 mg/dL in females) or specific drug treatment; (3) high blood pressure (≥ 130x85 mmHg) or medication treatment and (4) raised fasting plasma glucose (≥ 100 mg/dL) [39].

The Japan Society for the Study of Obesity (JASSO) criteria for MetS was used in Hishikawa et al. (2016) [20, 40]. The diagnosis of MetS required the presence of abnormal waist circumference (≥85 cm for males and ≥90 cm for females) and 2 or more of the following: (1) raised triglyceridemia (≥150mg/dL) or low HDL levels (<40 mg/dl); (2) high blood pressure (≥ 130x85 mmHg) and (3) hyperglycemia (≥110 mg/dL) [40].

### Cognitive assessment

Cognitive evaluation was limited to results on the Mini-Mental State Examination (MMSE) in 6 of the studies [14, 24, 27, 28, 35, 37]. Three of the studies used the 3MS, a modified-version of MMSE proposed by Teng & Chui (1987) [19, 32, 33, 41]. Other papers applied brief screening tests as unique measurements of cognitive performances: Del Brutto et al. (2016) [31] used the Montreal Cognitive Assessment (MoCA) and Laudisio et al. (2008) [23] chose the Hodkinson Abbreviated Mental Test. Other tasks used as single measurements of cognitive functions: the Digit Symbol Substitution Test [36] and the Clock Drawing-Test [16].

More extensive sets of cognitive tasks were performed in other studies [13, 14, 17, 18, 20–22, 25, 26, 29, 30, 32, 34].

The design of the included studies, the sample sizes, the follow-up durations and the list of cognitive tests are described on Table 1.

**Table 1:** Overview of studies on metabolic syndrome in the elderly.

Author, year, country	Type of study	n	Age (m±sd)	MetS criteria	Cognitive Assessment	Other variables	Follow-up duration
Yaffe, 2004, USA	L	2,632	73.6±2.9	NCEP-ATPIII	3MS	IL-6, C-reactive protein	5 years
Van den Berg, 2007, Netherlands	L	562	85 to 90*	NCEP-ATPIII	MMSE, Stroop test, Letter digit-coding test, 12 word-learning test	-	5 years
Yaffe, 2007, USA	L	1,624	70.5±7.0	NCEP-ATPIII	3MS, Delayed Word-list recall	C-reactive protein	1 year
Komulainen, 2007, Finland	L	101	63.7±2.8	NCEP-ATPIII	MMSE, STROOP test, Word Recall Test, Letter-Digit Substitution Test	-	12 years
Roriz-Cruz, 2007, Brazil	CS	422	68.1±1.4	NCEP-ATPIII	MMSE, Executive tasks from the Tokyo Metropolitan Institute of Gerontology (TMIG) scale	-	-
Dik, 2007, Netherlands	CS	1,183	75.1±6.4	NCEP-ATPIII	MMSE, RAVLT, Coding test, Raven Matrices	α1-antichymotrypsin, C-reactive protein	-
Laudisio, 2008, Italy	CS	353	79.0±5.5	NCEP-ATPIII	Hodkinson Abbreviated Mental Test	C-reactive protein	-
Lee, 2010, Korea	CS	2,944	72.1±6.7	NCEP-ATPIII	MMSE	-	-
Raffaitin, 2011, France	L	7,097	73.4±4.9	NCEP-ATPIII	MMSE, Isaacs Set Test, Benton Visual Retention Test	APOE4	4 years
Katsumata, 2012, Japan	L	148	85.0±3.2	NCEP-ATPIII	MMSE, phonemic VF, Scenery Picture Memory Test	-	3 years
Viscogliosi, 2012, Italy	CS	159	69.8±4.8	NCEP-ATPIII	MMSE	C-reactive protein	-
Liu, 2013, China	L	338	82.4±4.2	NCEP-ATPIII, IDF	MMSE	-	1 year
Shigaeff, 2013, Brazil	CS	49	73.9±5.9	NCEP-ATPIII	TMT A and B, Digit Span, RAVLT, Clock Drawing Test, Cubes, Boston Naming Test, Verbal Fluency	-	-
Watts, 2013, USA	L	148	74.4**	NCEP-ATPIII	MMSE, Wechsler Memory Scale–Revised Logical Memory tests, Free and Cued Selective Reminding Task, Wechsler Adult Intelligence Scale letter–number sequencing, digit symbol, and Stroop Test	Glucose tolerance test and serum insulin	2 years
Rouch, 2014, France	L	1,011	65.6±0.8	NCEP-ATPIII	Free and cued selective Reminding test, Digit symbol substitution test, Digit span, TMT A and B, Baddeley dual task, Stroop test, Verbal Fluency, WAIS similarities task.	-	10 years
Harrison, 2015, UK	L	845	85***	NCEP-ATPIII	MMSE, Cognitive Drug Research, Word Recognition Task	C-reactive protein	5 years
Viticchi, 2015, Italy	CS	162	72.4±4.2	NCEP-ATPIII	MMSE	Breath-Holding Index	-
Viscogliosi, 2015, Italy	CS	80	80.4±5.3	NCEP-ATPIII	MMSE, Babcock Short Story Recall, Clock Drawing test	C-reactive protein, Fazekas scale	-
Liu, 2015, China	CS	2,102	72.2 ±6.6	NCEP-ATPIII	MMSE	-	-
Ghosh, 2015, India	CS	109	69.28±6.72	IDF	3MS	C-reactive protein	-
Viscogliosi, 2016, Italy	L	104	80.2±5.5	NCEP-ATPIII	Clock Drawing test	Fazekas scale	10–12 months
Del Brutto, 2016, Ecuador	CS	212	69.2 ± 7.2	IDF	MoCA	-	-
Tsai, 2016, USA	CS	2,252	70.7±7.6	NCEP-ATPIII	Digit symbol substitution test	-	-
Hishikawa, 2016, Japan	CS	968	79.8 ± 8.0	JASSO	MMSE, Hasegawa Dementia Score- Revised FAB, MoCA, ‘Ryokansan’ (Ohtsu Computer Corp., Otsu, Japan).	Reactive hyperemia index, Fazekas scale	-
Viscogliosi, 2017, Italy	L	195	76.1 ±3.1	NCEP-ATPIII	MMSE	APOE4	10 years

CS = cross-sectional, L = longitudinal, n = sample size, m = mean, sd = standard deviation, MetS = metabolic syndrome, WHO = World Health Organization, NCEP-ATPIII = National Cholesterol Education Program- Adult Treatment Panel III, EGIR = European Group for the Study of Insulin Resistance, ACE = American College of Endocrinology, IDF = Internal Diabetes Federation, JASSO = Japan Society for the Study of Obesity; SE = standard error, HbA1 = glycosylated hemoglobin.

*Patients were followed annually from 85 to 90 years old.

** Study did not acknowledge the SD or the age range of the participants.

*** Assessments started in 2006 for patients born in 1921 (85 years old).

### Other clinical and laboratory variables

Biomarkers of high inflammation were measured in studies, such as: Serum α1-antichymotrypsin, C-reactive protein, interleukin-6 (IL-6), homocysteine and fibrinogen [13, 14, 16, 23, 26, 32, 33] (Table 1).

APOE4 carriers were identified in two articles [25, 27]. Indexes of endothelial function (reactive hyperemia index) and cerebral vasoreactivity to hypercapnia (the Breath-Holding Index) were applied in studies [20, 35]. The severity of white-matter lesions in brain structural MRI was measured through the Fazekas visual scale in three of the papers [15, 16, 20]. Intravenous glucose tolerance test and serum insulin were measured in one study [18].

### Metabolic syndrome, cognitive performance and risk of dementia

Patterns of baseline cognitive performances in non-demented elderly subjects with and without MetS varied largely among studies. In some of them, the presence of MetS was associated with lower scores in screening tests (namely MMSE and 3MS) compared to non-MetS group [13, 19, 22, 25, 32, 35, 37]. Impairments in specific cognitive functions were also associated with the diagnosis of MetS in both demented and non-demented subjects, such as sustained and alternating attention, processing speed, language, executive function, working memory and episodic memory [13, 17, 20, 22, 25, 34]. Presence of MetS inversely correlated with scores in the clock drawing test, but no association with a logical memory task was found [15]. However, multivariate analysis indicated that correlation between MetS and performances in episodic memory and executive function tasks remained significant even after controlling for sex, educational level, anxiety, depressive symptoms, and tobacco use [34]. Number of MetS components was associated with decrease in the MMSE [14, 22, 37], the word-recall test [30] and in the digit symbol substitution test scores [36] in studies.

The diagnosis of MetS was not associated with the presence of cognitive impairments in other sources. Performances in the MMSE, the 3MS and the MoCA did not differentiate subjects with and without MetS, regardless of age, in six of the articles [20, 21, 26, 29, 31, 33]. Computerized tasks assessing long-term memory, decision-making and attention did not distinguish AD subjects with and without MetS [20]. No significant group difference in cognitive performance was detected when adjustments for age, sex, APOE4 status and educational level were made in one paper [24].

Longitudinal impact of MetS over cognition produced divergent results. Declines in cognitive parameters (MMSE, 3MS, clock drawing test scores, besides visual working memory and episodic memory tasks) were greater in the follow-up of MetS compared to non-MetS participants in studies, although magnitude of change over time suffered substantial variations among articles [16, 19, 25, 27, 30, 33]. Moreover, significant risk was associated with combination of MetS and high inflammation in studies (RR = 1.66 (95% CI 1.19–2.32) [33]; and β = -0.46, p = 0.004 [32]). Nevertheless, other studies did not find significant cognitive change over time associated with MetS [18, 26, 29]. In fact, in subjects with AD, MetS was associated with better cognitive performances on attention and verbal memory at the two-year follow-up [18].

Effects of MetS on cognitive performances of older-old individuals were assessed in other studies. Multivariate analyses confirmed that MetS conferred increased risk for cognitive impairment for those below 80 years old, but not for older-old subjects [37]. Interestingly, one study which assessed subjects with 85 years old indicated an association between baseline high blood pressure and better performances in attention and episodic memory tasks compared with those with normal pressure [26]. Diagnosis of MetS showed a protective effect for cognitive decline among men aged 75 years or older with intact cognition in a year of follow-up [28]. Likewise, significant decelerated decline on the MMSE, the Stroop Test and the Letter Digit Coding Test were associated with diagnosis of MetS on individuals from 85 to 90 years old [21].

### Individual MetS components and cognitive performance

Analysis of the individual effects of metabolic disturbances over cognitive performances showed that hyperglycemia was most consistently associated with poor cognitive abilities and with the prevalence of MCI [13, 14, 33, 37] compared with other vascular risk factors. Although MMSE scores and risk for MCI were significantly related with central obesity, high blood pressure and hyperglycemia, after adjusting for age, sex, education, marriage status, smoking, alcohol drinking, physical activity, body mass index, family history of dementia and use of medication, only hyperglycemia remained significantly correlated with MMSE scores and diagnosis of MCI [37]. MMSE score negatively correlated with fasting hyperglycemia in an urban Chinese community sample; in addition, 2-hour plasma glucose levels were negatively associated with MMSE in females (but not in males) from the same sample [37]. High fasting glucose significantly correlated with episodic memory, cognitive speed and sustained attention in studies [33, 34, 36]. Similarly, glycosylated hemoglobin levels were associated with decreased scores in a visual memory task over time [29] and higher insulin at baseline predicted greater declines in verbal memory and a trend for greater declines in attention over the two year follow up in healthy older subjects [18]. Although higher insulin has predicted better verbal memory in early AD individuals, at the two-year follow-up, higher glucose predicted more rapid declines in attention after the same period [18]. Diagnosis of diabetes (but not high fasting glucose) was associated with decline in visual working memory and verbal fluency tasks after 4 years in a multivariate analysis controlling for age, gender, educational level, location of assessment, baseline cognitive scores, APOE4 status, smoking habit, cardiovascular disease and presence of depressive symptoms [25].

More heterogeneous findings regarding the effects of other MetS components on cognition were reported. Systolic blood pressure was negatively correlated with MMSE [37] and clock drawing test scores in studies [16]. Among female participants, diastolic blood pressure also negatively correlated with MMSE [37]. Performance in the digit-symbol substitution test inversely correlated with the presence of high blood pressure [36]. Also, systemic arterial hypertension at baseline was associated with decline in visual working memory, but in the MMSE score, after 4 years [25]. However, no significant relationship between high blood pressure and cognitive status was established in other studies [13, 33].

Studies which assessed impact of lipid blood levels over cognition evidenced that low HDL correlated with reduced MMSE scores, decreased cognitive speed and poorer executive functions [13, 25, 34]. Hypertriglyceridemia was associated with worse episodic memory at baseline [33] and lower MMSE and visual working memory scores after 4 years [25]. Moreover, effects of high triglycerides and low HDL showed significance exclusively in APOE4 carriers, after adjusting for age, gender and education, in one study [24]. Central obesity appeared as a risk factor for cognitive decline in males over 75 years old after one year of follow-up in one paper [28], but this association was not significant in both cross-sectional and longitudinal multivariate analysis with older samples of both genders and without age limits [13, 22, 25, 33]. Finally, central obesity, but not high triglycerides or low HDL levels, correlated with poor performances in the digit-symbol substitution test [36].

### Inflammation, cognitive decline and other variables

Presence of elevated C-reactive protein was associated with dementia in subjects with MetS, which was not observed in those without MetS [19]. Similarly, subjects with MetS and high C-reactive protein suffered greater cognitive decline in the follow-up than controls and subjects with MetS without high inflammation markers [32, 33]. Controversially, high C-reactive protein level was not associated with presence of MetS or with change in MMSE performance over time in one study with an 85-year-old sample [26]. Severity of white-matter hyperintensities was not associated with diagnosis of MetS in subjects with AD [20]. Reactive hyperemia index was significantly lower in AD subjects with MetS than in those with no MetS [20]. The Breath-holding index was associated with lower MMSE, independently of MetS diagnosis [35].

Table 2 summarizes the associations between MetS (and its components) and the risk for cognitive decline in the longitudinal studies. Statistical combination of data was not performed because of substantial heterogeneity in the methods among the included articles.

**Table 2:** Metabolic syndrome (and its components) and risk for cognitive decline in longitudinal studies.

Study, year	Duration of follow-up (years)	Outcome variables	Risk associated with MetS	Risk associated with high BP	Risk associated with high TG	Risk associated with low HDL	Risk associated with high FG	Risk associated with central obesity
Yaffe, 2004	4	3MS	RR = 1.66 (95% CI 1.19–2.32)*	-	-	-	-	-
Komulainen, 2007	12	MMSE	n.s.	n.s.	n.s.	n.s.	n.s.	n.s.
		Word Recall Test	OR = 2.47 (95% CI: 1.05–5.85	n.s.	n.s.	z-score = -1.29 (95% CI: –2.51 to –0.06)	n.s.	n.s.
		Stroop Test/ Letter-Digit Substitution Test	n.s.	n.s.	n.s.	n.s.	n.s.	n.s.
Van den Berg, 2007	5	MMSE	β = -0.66 (SE = 0.05)	n.s.	n.s	n.s.	β = 0.24 (SE = 0.08)	β = 0.25 (SE = 0.07)
		Stroop Test	n.s.	-	-	-	-	-
		Letter-Digit Coding	β = -0.52 (SE = 0.07)	-	-	-	-	-
		Word-Learning-Immediate	β = 0.90 (SE = 0.08)	-	-	-	-	-
		Word-Learning-Delayed	β = 0.44 (SE = 0.04)	-	-	-	-	-
Yaffe, 2007	1	3MS	β = 0.46*	n.s.	n.s.	n.s.	n.s.	n.s.
		Word-List Recall-Delayed	n.s.	n.s.	n.s.	n.s.	β = -0.12	n.s.
Raffaitin, 2011	4	MMSE	HR = 1.20 (95% CI 1.06–1.36)	n.s.	n.s.	HR = 1.19 (95% CI 1.03–1.38)	n.s.	n.s.
		Verbal Fluency	n.s.	n.s.	n.s.	n.s.	n.s.	n.s.
		Benton Visual Retention Test	HR = 1.13 (95% CI 1.01–1.26)	HR = 1.17 (95% CI 1.03–1.32)	n.s.	n.s.	n.s.	n.s.
Katsumata, 2012	2.33	MMSE	n.s.	n.s.	n.s.	n.s.	-	n.s.
		Phonemic Verbal Fluency	n.s.	n.s.	n.s.	n.s.	-	n.s.
		Scenery Picture Memory Test	n.s.	n.s.	n.s.	n.s.	-	n.s.
Liu, 2013	1	MMSE	OR = 0.15 (95% CI: 0.33–0.64	-	-	-	-	-
Watts, 2013	2	MMSE	n.s.	n.s.	n.s.	n.s.	n.s.	n.s.
		Digit-Symbol (WAIS)	n.s.	n.s.	n.s.	n.s.	β = −0.335**	n.s.
		Verbal Memory (Wechsler-Revised)	n.s.	n.s.	n.s.	n.s.	n.s.	n.s.
Rouch, 2014	10	Free and Cued Selective Reminding test	OR = 1.77 (95% CI 1.16–2.69)	n.s.	n.s.	n.s.	OR = 1.53 (95% CI 1.01–2.31)	n.s.
		Executive function battery	OR = 1.91 (95% CI 1.05–1.19)	n.s.	n.s.	OR = 2.60 (95% CI 1.68–4.02)	n.s.	n.s.
Harrison, 2015	5	MMSE	n.s.	n.s.	n.s.	n.s.	n.s.	n.s.
	3	Cognitive Drug Research: Focused attention test	n.s.	n.s.	n.s.	n.s.	n.s.	n.s.
		Cognitive Drug Research: sustained attention test	n.s.	n.s.	n.s.	n.s.	n.s.	n.s.
		Word recognition task	n.s.	n.s.	n.s.	n.s.	n.s.	n.s.
Viscogliosi, 2016	1	Clock Drawing Test	β = −0.327, t = −3.059, df = 96	β = −0.436, t = −4.902, df = 96***	n.s.	n.s.	n.s.	n.s.
Viscogliosi, 2017	10	MMSE	OR = 1.38 (95% CI: 1.20–1.58)	n.s.	n.s.	n.s.	n.s.	n.s.

MetS = Metabolic syndrome; BP = high blood pressure; TG = triglycerides; HDL = high-density lipoprotein; FG = fasting glucose; MMSE = Mini-Mental State Examination; 3MS = modified Mini-Mental State Examination, n.s. = not significant; SE = standard error; HR = hazard ratio

*Only significant for subjects with high levels of C-reactive protein (≥2.0 mg/L) and IL-6 (≥2.0 pg/mL).

**Only significant for early Alzheimer’s disease subjects.

***Only significant for Systolic Blood Pressure.

## Discussion

Current available data on the effects of metabolic risk factors—whether clustered as MetS or individually—on cognition of older subjects is highly inconclusive and based on results from studies with potential biases. Although diagnostic criteria for MetS applied in the studies followed recognized recommendations from experts’ committees (NCEP-ATP III, IDF and JASSO), multiple minor changes in some of their original aspects were undertaken by many authors, which may have accounted, to some extent, for divergent results. In fact, different methods for diagnosing glucose metabolic disturbances may have led to the selection of heterogeneous groups of participants, as suggested by some studies. For instance, a previous meta-analysis indicated that HbA1c levels may be strongly correlated with postprandial plasma glucose, but not with fasting glucose [42]. In addition, a review stated that concentration of fructosamine may fluctuate in response to a variety of systemic disorders and that the current method for measuring this marker still needs to be better standardized [43]. Moreover, elevating the cutoff for systemic arterial hypertension for ≥ 160x90 mmHg as adopted by Dik et al. (2007) [13], may have determined the selection of individuals with more severe vascular disorders and consequently with higher levels of inflammation markers. Hence, findings from this study may not be comparable with those of the other studies, which classified subjects with high blood pressure through the original NCEP-ATP III criterion (≥ 130 x 85 mmHg). Although central obesity showed little if any impact over cognition of older subjects, the adoption of body-mass index instead of waist circumference [21, 22, 29, 35], may have accounted for lower accuracy in the detection of this condition, as suggested by a previous survey [44]. Furthermore, the assumption of pharmacological treatment as alternative criteria for the identification of systemic arterial hypertension, hyperglycemia and dyslipidemia has inhibited the use of the raw values for blood pressure, fasting blood glucose and lipid levels on regression analyses. To avoid the mitigating effect of medications over those rates, authors have chosen to refer to those conditions as dichotomous variables (presence/absence), which has not allowed the estimation of the effects of different degrees of severity of metabolic disturbances over cognition [13, 23, 29, 36].

Moreover, cognitive assessment methods in the studies were overall concise, consisting of the application of abbreviated sets of cognitive tests. Noteworthy, 48% of the selected articles applied single screening tools to evaluate cognition (Table 1). The need for harmonizing cognitive testing for longitudinal studies on aging and dementia has been addressed by international working groups and recommendations for a comprehensive protocol have been published [45, 46].

In the present review, evidence of the influence of MetS on cognitive decline in elderly population was vastly inconsistent. Glucose metabolism disturbances, whether represented by fasting hyperglycemia, high insulin, increased fructosamine, elevated HbA1c or diagnosis of diabetes mellitus, may have been more steadily associated with cognitive impairments in older subjects than other components of MetS. In fact, mounting knowledge has suggested a common molecular defect linking type 2 diabetes and Alzheimer’s Disease, which resulted in defective insulin signaling and chronic inflammation in the brain tissue [47]. Evidence linking high glucose blood levels at midlife and cognitive decline in elderly population suggested that long-term brain insults associated with hyperglycemia might play an important role for the development of dementia [48, 49]. On the other hand, high triglyceride levels showed no significant impact on cognition of older individuals in the studies. Benefits of statin-therapy for cardiovascular prevention have long been objects of dispute, but recent surveys did not support the use of these drugs for the treatment of dyslipidemia in older adults [50].

Remarkably, data on MetS in oldest-old persons (≥75–80 years old) appeared to uniformly indicate a protective effect of metabolic disorders for cognitive functioning of those subjects [21, 26, 28, 37]. Similar findings were consistently reported in previous studies and interpretation for them has been challenging and mostly speculative [51, 52]. It has been suggested that the role of metabolic conditions may change over the course of life and that thresholds used for young adults could not be appropriated for the older population. Adaptive changes associated with aging may result in altered vasoreactivity, body composition and metabolism, which instead of harmful could be beneficial for older adults’ health and cognitive function. For instance, some studies stated that body mass index below 25 may be a risk factor for cognitive decline and mortality in elderly subjects [53, 54]. Likewise, late-life hypertension in the oldest-old may prevent the onset of dementia, as suggested by a previous study, which applied the cutoff recommended by the Seventh Report of the Joint National Committee on Prevention, Detection, Evaluation, and Treatment of High Blood Pressure for the detection of the abnormality (≥140x90 mmHg) [51]. As a result, differential targets for blood pressure in the oldest-old have been suggested by some groups of experts [55–57], but large longitudinal studies are still needed to verify the validity of this change. Moreover, other relevant factors, such as alcohol and tobacco use, sleep disturbances, sedentary lifestyle, history of stress-related disorders and unhealthy dietary habits, are not accounted within the MetS construct and their importance for cognitive decline in the elderly ought to be furtherly investigated [58–61].

Other limitations of the present review ought to be acknowledged. Issues regarding the recruitment processes of the samples might have played a confounding effect when analyzing pooled data from the selected articles. Substantial methodological heterogeneity–for instance, limiting the inclusion of participants according to age, gender, ethnic group and clinical status criteria (exclusion of subjects with: history of coronary arterial disease in Viscogliosi et al., 2012 [14]; history of myocardial infarction or other heart disease in Rouch et al., 2014 [34]; diabetes mellitus in Viscogliosi et al., 2015 [15] etc.)—did no allow the statistical combination of cognitive findings across the studies. Consistently, a previous meta-analysis which examined the association between MetS and cognitive function in subjects of all ages failed to detect a significant relationship and yielded some degree of heterogeneity of the individual cognitive estimates [62]. In addition, risk of biases within each individual study should be considered: significant differences between groups with and without MetS concerning to age, years of education, gender and ethnic group, as previously mentioned, may have interfered with the interpretation of the results. Likewise, studies using convenience samples from medical or nurse care facilities may not represent the general population [14–17,19,20]. This choice was motivated by the need to include more data from middle income countries [17–19], considering that 76% of the selected studies were conducted in developed nations. Furthermore, since most articles presented predominantly positive results, the risk for publication bias cannot be excluded.

In conclusion, the authors state that current evidence is insufficient to confirm whether MetS in a risk factor for cognitive decline in the older population. Future studies evaluating MetS should consider age-related discrepancies within the older range regarding the impact of each component over cognition. Prior to that, however, researchers should address the need for harmonizing the diagnostic criteria for MetS in elderly population, including the definition of optimal metabolic targets in this population.

## Supporting information

S1 Table PRISMA 2009 checklist. (DOCX)
